# Molecular Genetics of *FAM161A* in North American Patients with Early-Onset Retinitis Pigmentosa

**DOI:** 10.1371/journal.pone.0092479

**Published:** 2014-03-20

**Authors:** Giulia Venturini, Silvio Alessandro Di Gioia, Shyana Harper, Carol Weigel-DiFranco, Carlo Rivolta, Eliot L. Berson

**Affiliations:** 1 Department of Medical Genetics, University of Lausanne, Lausanne, Switzerland; 2 The Berman-Gund Laboratory for the Study of Retinal Degenerations, Harvard Medical School, Massachusetts Eye and Ear, Boston, Massachusetts, United States of America; Telethon Institute of Genetics and Medicine, Italy

## Abstract

Retinitis pigmentosa (RP) is a hereditary disease that leads to the progressive degeneration of retinal photoreceptor cells and to blindness. It is caused by mutations in several distinct genes, including the ciliary gene *FAM161A*, which is associated with a recessive form of this disorder. Recent investigations have revealed that defects in *FAM161A* represent a rather prevalent cause of hereditary blindness in Israel and the Palestinian territories, whereas they seem to be rarely present within patients from Germany. Genetic or clinical data are currently not available for other countries. In this work, we screened a cohort of patients with recessive RP from North America to determine the frequency of *FAM161A* mutations in this ethnically-mixed population and to assess the phenotype of positive cases. Out of 273 unrelated patients, only 3 subjects had defects in *FAM161A*. A fourth positive patient, the sister of one of these index cases, was also identified following pedigree analysis. They were all homozygous for the p.T452Sfx3 mutation, which was previously reported as a founder DNA variant in the Israeli and Palestinian populations. Analysis of cultured lymphoblasts from patients revealed that mutant *FAM161A* transcripts were actively degraded by nonsense-mediated mRNA decay. Electroretinographic testing showed 30 Hz cone flicker responses in the range of 0.10 to 0.60 microvolts in all cases at their first visit (age 12 to 23) (lower norm  =  50 μV) and of 0.06 to 0.32 microvolts at their most recent examination (age 27 to 43), revealing an early-onset of this progressive disease. Our data indicate that mutations in *FAM161A* are responsible for 1% of recessive RP cases in North America, similar to the prevalence detected in Germany and unlike the data from Israel and the Palestinian territories. We also show that, at the molecular level, the disease is likely caused by FAM161A protein deficiency.

## Introduction

Retinitis pigmentosa (RP) is a group of hereditary degenerative diseases of the retina displaying a significant phenotypic and genotypic variability and characterized by the progressive decrease of rod and cone photoreceptor function. Patients typically report loss of night vision during adolescence, loss of mid and far peripheral field in young adulthood, and loss of central vision in later life. Characteristic clinical features are elevated final dark adaptation thresholds and reduced and delayed full-field electroretinogram (ERG) signals, measuring the electrical response of the retina to flashes of light [1]. At present, according to the RetNet database (https://sph.uth.edu/retnet/), over 60 genes have been associated with nonsyndromic RP, which account for 50-60% of patients with this condition [2], [3].

Mutations in *FAM161A* have been found to represent the cause of *RP28*-associated autosomal recessive RP (arRP) in the initial Indian family in which the RP28 locus was mapped [4], as well as in a cohort of German patients [5]. Frequent nonsense mutations in *FAM161A* were also identified in patients from Israel and the Palestinian territories [6]. FAM161A is evolutionarily conserved in vertebrates [5], [6] and, despite having multiple splicing variants, only two of them produce stable mRNA transcripts. The main splicing variant contains 6 exons and encodes a protein of 76 kDa, while the second variant contains a supplementary 168 bp in-frame exon between exons 3 and 4 [5], [6]. The protein is mainly expressed in the retina and in the testis and localizes in photoreceptor cells during mouse retinal development [5], [6]. More specifically, FAM161A localizes at the base of the photoreceptor connecting cilium and at the ciliary basal body [7], [8]. Patients with *FAM161A* mutations have been reported to have symptoms ranging from an atypically late-onset form of RP, detected in the German cohort mentioned above, to an early-onset manifestation of the disease, as observed in Israeli and Palestinian individuals [5], [6]. Unexpectedly, despite these clinical differences, all patients who have been ascertained so far carry the same class of mutations, leading to premature stops of the open reading frame.

Here we report results of our mutational screening of *FAM161A* in a cohort of patients with autosomal recessive RP from North America, with the aim of assessing the mutation spectrum in this population and investigating any possible genotype/phenotype correlation.

## Methods

### Patients and Controls

This research was carried out in accordance with the tenets of the Declaration of Helsinki and was approved by the Institutional Review Boards of the University of Lausanne and of Harvard Medical School and the Massachusetts Eye and Ear, where the blood was collected and the patients were followed. Written informed consent was obtained from patients who participated in this study or from their legal guardians before they donated 10-30 ml of their blood for research.

All patients were evaluated with respect to best-corrected Snellen acuities, kinetic visual fields on the Goldmann perimeter with a V-4e white test light, final dark adaptation thresholds with an 11° white test light in a Goldmann-Weekers dark adaptometer, and full-field cone ERGs in response to 30 Hz white flicker obtained with narrow bandpass filtering and computer averaging, as described previously [9]. After dilation, all had a slit-lamp examination, fundus evaluation with direct and indirect ophthalmoscopy, and fundus photography.

DNA from peripheral blood leukocytes was extracted from 273 unrelated patients with arRP from North America, representing an ethnically-mixed population. Controls included 95 individuals with no history of retinal degeneration and 80 subjects with normal ERGs. In addition, DNA samples from 95 ethnically-matched healthy individuals were purchased from the Coriell Institute Repository. Initial screening of *FAM161A* sequence was performed on whole-genome amplified DNA (REPLI-g Mini Kit, Qiagen). All samples that were found to be positive for DNA changes were then PCR-amplified and sequenced a second time, starting from original DNA.

### Mutation Screening

Primer pairs for individual exons and relevant intron boundaries were designed using the CLCbio Genomics Workbench (Table S1). Amplifications by PCR were performed in 25 μl reactions containing 20 ng genomic DNA, 1× GoTaq buffer, 1.2 mM MgCl_2_, 0.1 mM dNTPs, 0.4 μM of each primer, and 0.01 U/μl of GoTaq polymerase (Promega). Amplification conditions were: an initial step at 95°C for 2 minutes, 35 cycles of denaturation at 94°C for 30 seconds, annealing according to primers' melting temperature for 30 seconds, and extension at 72°C for 1 minute. Before the end of the reaction, a final extension step at 72°C for 5 minutes was performed.

Sequencing reactions were carried out by Sanger sequencing, after purification of PCR products (ExoSAP-IT, USB), by using 1 μl of 3.3 μM sequencing primer (Table S2) and 0.5 μl of BigDye Terminator v1.1 (Applied Biosystems). The products of these sequencing reactions were run on an ABI-3130XLS sequencer (Applied Biosystems).

### Cell Culture and Drug Treatment

The Epstein-Barr virus immortalized lymphoblastoid cell lines that were used in this study were derived from two affected patients (003-161 and her sibling 012-001), and were cultured as previously described [10]. Normal control cell lines were purchased from the Coriell Cell Repository. RNA extraction, cDNA synthesis, and treatment of cells with cycloheximide was performed as previously reported [11].

*FAM161A* primers for cDNA amplification were 5′-ggaagaaacgaaaagaatgg-3′ and 5′–ttctcgttggtattctctcatcc-3′, yielding a 1,115 bp product. 18S ribosomal RNA was used as a housekeeping gene for this analysis, and primers for its cDNA amplification were 5′-cggctaccacatccaaggaa-3′ and 5′–gctggaattaccgcggct-3′ (resulting in a 187 bp product).

### RNA Ligase Mediated Rapid Amplification of cDNA Ends (RLM-RACE)

To perform RLM-RACE we used the FirstChoice RLM-RACE kit (Invitrogen), following the manufacturer's instruction. One microgram of total RNA from human retina (Clontech) was used to perform 3′UTR RLM-RACE, and ten micrograms of total RNA from human retina (Clontech) and from retinoblastoma cell line Y79 were used to perform 5′UTR RLM-RACE.

## Results

### Molecular Genetic Analysis

We screened *FAM161A* in 273 unrelated patients with arRP from North America, and in 270 ethnically matched healthy individuals. In three of these patients we identified the null mutation p.Thr452SerfsX3, which was previously reported as a founder mutation in an Israeli Jewish population [6]. Following the pedigree analysis of these three index patients, we identified an additional affected family member (012-001, sister of 003-161), who was also found to be positive for the same mutation. All four patients were homozygous for p.Thr452SerfsX3 (Table 1). All controls were negative for this DNA change.

**Table 1:** Variants identified in this study.

Patient ID	Nucleotide change	Predicted effect	Polyphen^a^	SIFT^b^	MutPred^c^	PMut^d^	Controls frequency (alleles)	Reference of the variation
003–327	c.1113 C>G/+	p.Asp371Glu/+	benign	tolerated	benign	neutral	0/540	This study
003–154, 003–217, 121–847, 121–050, 121–543	c.1133 T>G/+	p.Leu378Arg/+	probably damaging	tolerated	disrupted	pathological	2/540	Langmann et al. [5]
003–218	c.1153 C>G/+	p.Gln385Glu/+	probably damaging	tolerated	benign	neutral	2/540	Langmann et al. [5]
003–189	c.1391 A>G/+	p.His464Arg/+	benign	tolerated	benign	pathological	0/540	This study
003–161, 003–257, 121–385, 012–001	c.1355_6delCA/c.1355_6delCA	p.Thr452SerfsX3/p.Thr452SerfsX3	confirmed mutation				0/540	Bandah-Rozenfeld et al. [6]

**Figure 1:**
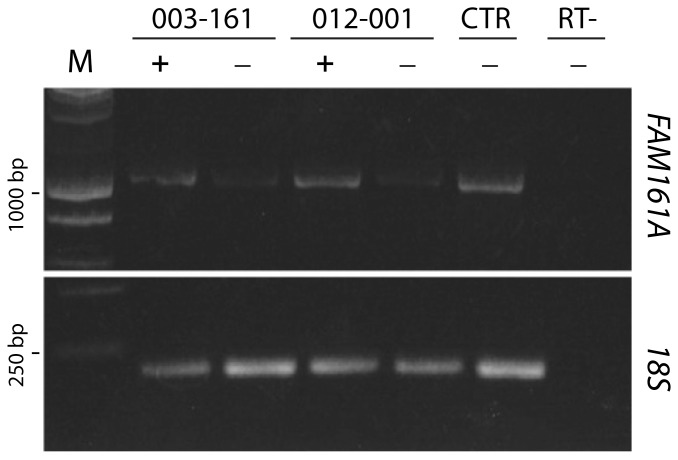
Mutation c.1355_6delCA (p.T452SfsX3) produces transcripts with a premature stop codon that are targets for NMD degradation. The image shows an agarose gel on which six RT-PCR products were run: two from patients 003-161 and 102-001, respectively, one from a control cell line (CTR), and one from the same control cell line, following a cDNA synthesis reaction performed without the addition of the reverse transcriptase enzyme (RT-). Presence of cycloheximide in cell cultures is indicated with "+", its absence with "−". M, DNA molecular size marker; 18S, RT-PCR loading control (18S rRNA); 1000 bp and 250 bp, bands of the marker corresponding to these specific sizes, respectively.

+, wild-type allele.

a http://genetics.bwh.harvard.edu/pph2/.

b http://sift.jcvi.org/.

c http://mutpred.mutdb.org/

d http://mmb2.pcb.ub.es:8080/PMut/.

p.Thr452SerfsX3 is predicted to produce a transcript that could be a target for nonsense mediated mRNA decay (NMD) and therefore result in no protein product [12]. In order to test this hypothesis, we analyzed lymphoblastoid cell lines derived from the two siblings carrying this change (003-161 and 012-001). RT-PCRs of *FAM161A* mRNA from both cell lines, cultured in standard conditions, yielded a rather poor amplification product, indicative of mRNA degradation. Conversely, supplementation with the chemical NMD inhibitor cycloheximide reversed this phenomenon and allowed good amplification, confirming that p.Thr452SerfsX3 results in a null allele (Figure 1).

In our cohort we also found a few other rare variants, likely having no pathological relevance (Table 1). Variant p.Q385E (rs139266382; Minor Allele Frequency, or MAF<0.01) was previously identified as a heterozygous change in arRP patients and in healthy controls with a frequency of 2/400 alleles [5]. In our screening, we identified it in one patient and in 2 individuals from our control cohort. Variant p.H464R (rs201315315, MAF<0.01) was found as a heterozygous change in one arRP patient and in no healthy controls. Variant p.D371E was neither annotated in the dbSNP database [13] nor was found in the control cohort, but was found in a heterozygous state in one patient. However, it involved an amino acid residue that is not conserved across species and was predicted to result in a benign change by *in silico* analyses.

Variant p.L378R was previously identified in the heterozygous state in a cohort of German patients with recessive or isolated forms of RP and classified as a rare variant with uncertain pathogenicity [5]. It involves a highly conserved residue and is predicted to be deleterious by 3 out of 4 prediction programs (Polyphen, SIFT, MutPred, and PMut) [14]–[17]. Recently, it has been annotated in the dbSNP database (build 135/138, rs187695569), with a MAF of 0.003. We identified it in a heterozygous state in 5 out of 273 arRP patients and in 2 healthy individuals from the Coriell control cohort.

**Figure 2:**
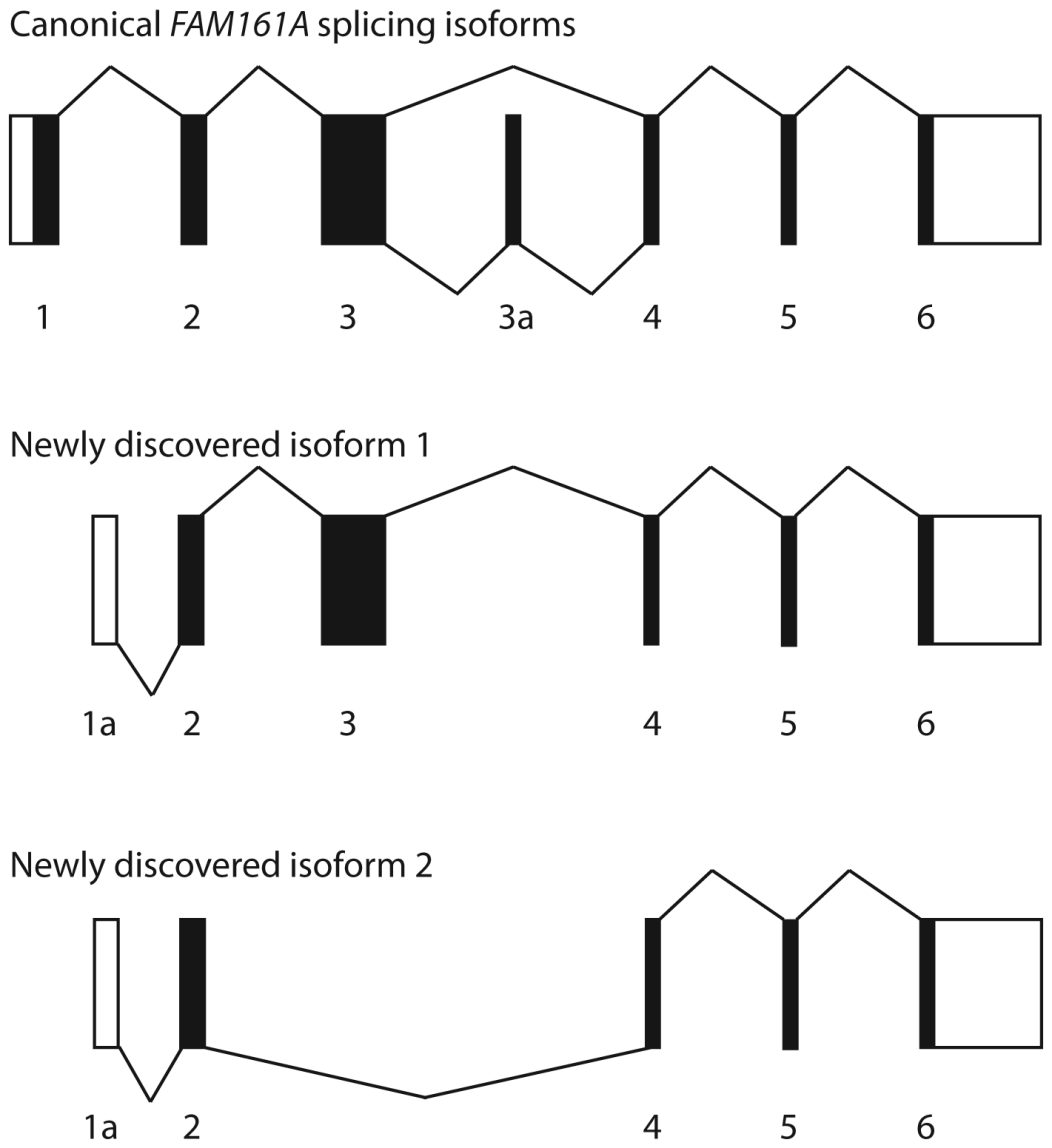
Schematic representation of alternative splicing events of *FAM161A* transcripts. Boxes represent exons, while lines represent splicing events. Coding regions are in black, noncoding regions are white. The canonical forms of *FAM161A* mRNA are presented in the top panel, as reference. The bottom two panels show newly-discovered splicing isoforms with an alternative 5′UTR in intron 1 (1a).

To test the hypothesis that heterozygous changes detected in our cohort of patients could in fact represent unrecognized mutations, we ascertained whether there were other non-annotated *FAM161A* exons in the human retina. We therefore performed 5′ and 3′ RLM-RACE PCR using pooled retinal RNA from different donors, and identified two additional transcripts with an alternative 5′UTR in intron 1, whose sequence corresponds to the annotated exon 2 of *FAM161A-003* isoform. In these transcripts, which were expressed in the retina at lower levels compared to the major isoforms, the translation-initiation codon may lie in exon 2 (Figure 2) [6]. Interestingly, one of them completely skipped the highly-conserved exon 3, where most *FAM161A* mutations have been so far reported. Furthermore, despite the absence of exon 1 and the skipping of exon 3, in both newly-discovered isoforms the original *FAM161A* open reading frame appeared to be preserved, possibly leading to translation of these transcripts. We therefore screened this alternative 5′UTR sequence in the patients, but we did not find any likely pathological variant.

### Clinical Evaluation of the Patients

**Figure 3:**
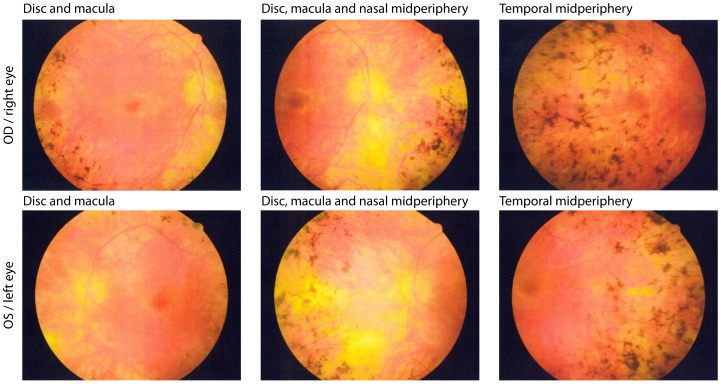
Fundus photographs from patient 121–385 (male, 43 years old). The patient shows the representative phenotype of this mutation with waxy pallor of the optic disc, retinal arteriolar attenuation, granularity of the macula and intra retinal pigment around the midperiphery in a bone spicule or clumped configuration.

Clinical evaluation at initial visits showed preserved visual acuity, constricted visual fields, and reduced but detectable 30 Hz cone ERGs in all 4 patients with known pathogenic mutations (Table 2). All had bone spicule pigment in the periphery (Figure 3). At follow up, 10 to 20 years later, each patient showed preserved acuity in at least one eye and cone ERGs comparable in amplitude to those at their initial visits. Because of a possible floor effect [9], further degeneration was difficult to quantify in these patients due to their small initial ERGs amplitudes.

**Table 2:** Clinical summary of patients with *FAM161A* mutations associated with retinitis pigmentosa.

Time of examination	Patient ID	Age	Sex	EO	VA^a^ OD	VA^a^ OS	ERG^b^ OD	ERG^b^ OS	VF^c^ OD	VF^c^ OS	DA^d^	Lens^e^ OD	Lens^e^ OS	Macula^f^ OD	Macula^f^ OS	Periphery^g^ OD	Periphery^g^ OS
First visit	003–161	12	F	Jewish (European)	20/40	20/30	0.10	0.10	1174	2016	3.0	–	–	–	–	+	+
First visit	003–257	21	F	Jewish (Moroccan)	20/50	20/40	0.10	0.10	108	76	3.0	–	+	–	–	+	+
First visit	121–385	23	M	Caucasian	20/20	20/20	0.24	0.60	2060	1795	3.0	+	+	–	–	+	+
First visit	012–001	12	F	Jewish (European)	20/40	20/40	0.10	0.10	78	78	3.0	+	+	–	–	+	+
Last visit	003–161	27	F	Jewish (European)	20/30	20/25	0.17	0.24	123	118	NA	–	–	–	–	+	+
Last visit	003–257	38	F	Jewish (Moroccan)	HM	20/60	0.06	0.10	NA	NA	4.0	+	+	AS	+	+	+
Last visit	121–385	43	M	Caucasian	20/20	20/30	0.22	0.32	69	65	4.0	PP	PP	+	+	+	+
Last visit	012–001	30	F	Jewish (European)	20/30	20/30	NA	NA	43	73	NA	+	+	–	–	+	+

a Visual Acuity: best corrected Snellen visual acuity.

b Electroretinograms: full field cone ERG amplitude in microvolts to 30HZ white light (lower norm  = 50 microvolts).

c Visual Field: Goldmann total field area to V-4e white test light (lower norm  =  11,399 degrees squared).

d Dark adaptation: final threshold in log units above normal after 45 minutes of dark adaptation.

e Lens: −, clear lens; +, central posterior subcapsular cataract.

f Macula: −, within normal limits; +, granular.

g Periphery: bone spicule or clumped pigment in one or more quadrants: +, present; −, absent.

Abbreviations: F, female; M, male; EO, ethnic origin; OD, right eye; OS, left eye; HM, hand motions; PP, pseudophakia; AS, atrophic scar; NA, not available.

## Discussion

Mutations in *FAM161A* were recently reported to cause autosomal recessive retinitis pigmentosa by two independent studies [5], [6]. However, despite mutations corresponded in all cases to DNA changes that lead to the premature termination of the reading frame, both prevalence and clinical severity of *FAM161A*-linked disease seem to be population-specific and/or dependent on the presence of other modifier genes. In Germany, this gene is mutated with a prevalence that is comparable to that of most arRP genes (1–2%) and is found in patients with a remarkably late-onset of the disease [5]. In Israel and in the Palestinian territories, *FAM161A* mutations account for about 12% of recessive RP cases, who have symptoms and signs of disease much earlier in their life [6].

Among 273 unrelated recessive patients screened in our study, only 3 were positive for *FAM161A* mutations, and in particular for p.T452Sfx3, indicating that this gene is rarely associated with RP in North America. Interestingly, p.T452Sfx3 was previously reported as a founder mutation in the Israeli and Palestinian populations [6], and all but one of our patients reported Jewish ancestry. Furthermore, from our study it emerged that a few patients carried rare heterozygous variants that were absent or had an allele frequency of less than 1% in the general population, and that had an uncertain functional significance.

Patients with *FAM161A* mutations clinically showed early-onset RP with relatively good acuity and very reduced cone ERGs. Follow-up examinations 10–20 years after the initial visit showed that all retained good acuity in at least one eye, 3 out of 4 showed further loss of visual field, and 1 showed further decline in the ERG. A floor effect may have occurred in those with less than or equal to 0.34 μV at their initial visit that could simulate stabilization or even improvement. Visual field areas to the V-4e white test light were all below normal. The phenotype was that of typical retinitis pigmentosa in all 4 cases. The long-term course of arRP associated with *FAM161A* mutations remains to be defined.

Finally, our analysis of *FAM161A* transcripts revealed that p.T452Sfx3 probably results in a functionally null allele, in view of the premature termination codon introduced by the mutation. Since all other pathogenic changes detected so far belong to this same class of mutations, it is likely that the disease is caused by deficiency of FAM161A protein, making the *FAM161A* gene a good candidate for gene replacement studies.

## Supporting Information

Table S1 **Primers for polymerase chain reaction amplification of** ***FAM161A*** **exons.**
(PDF)

Table S2 **Primers for Sanger sequencing of** ***FAM161A*** **exons.**
(PDF)
