# Supplementary material for: Molecular Genetics of FAM161A in North American Patients with Early-Onset Retinitis Pigmentosa
Source: PLoS One. 2014 Mar 20;9(3):e92479. doi: 10.1371/journal.pone.0092479 (PMC3961368; doi:10.1371/journal.pone.0092479)
Supplement: Table S1 — Primers for polymerase chain reaction amplification of FAM161A exons. (PDF) [file pone.0092479.s001.pdf]

Table S1. Primers for polymerase chain reaction amplification of *FAM161A* exons

Targeted Exon	Sense primer	Antisense primer	Annealing temperature (°C)	Amplification product (bp)
Exon 1	AGTGGATTTTGCGTGACTTTTG	AATAACGAAACACACCTGACAGAAC	59	728
Exon 2	CTCCCTCTCGTTCTTAAAATATCAC	TGGGTGGGTCAGAAAGAAAAGAC	59	668
Exon 3 (I)	GTAAAATGAAGGAGTCAAAGTGGA	CACCTAACCTTGTGTTTACACTTC	58	1359
Exon 3 (II)	GATTTAAAGCCAGACCCATTCCT	CCCAGCCAGGTAAAACTAGAAATC	56	768
Exon 3a	ATTCTGATTGGCTTAAAGTGG	TGGCTTTGAGGGAGATAGTTTC	62	807
Exon 4	GAGAATTAACCGGTATAGAAGAGGA	ATGATGAAGCCAACACAAACAACA	56	437
Exon 5	GGAGTGTAATTGTTGGTCATAGGGT	AAACGCTATAAAAGTGCCCATTG	53.5	969
Exon 6	ATACTTGCAGAGGGTTTGTTTAC	AACCACCAAAGACCAATACTTCTC	59	591

Abbreviations: bp, base pairs
