# Supplementary material for: Molecular Genetics of FAM161A in North American Patients with Early-Onset Retinitis Pigmentosa
Source: PLoS One. 2014 Mar 20;9(3):e92479. doi: 10.1371/journal.pone.0092479 (PMC3961368; doi:10.1371/journal.pone.0092479)
Supplement: Table S2 — Primers for Sanger sequencing of FAM161A exons. (PDF) [file pone.0092479.s002.pdf]

Table S2. **Primers for Sanger sequencing of *FAM161A* exons**

Targeted Exon	Sequencing primer
Exon 1	GTAGGGACTATGTGCACAGG
Exon 2	AAGCAGCATTTTGGATCAGTG
Exon 3 (I)	TACAAGGCAGAGGAGATGA
Exon 3 (II)	CAACATAAACTCCACAGAGC
Exon 3a	ATTCTGATTGGCTTAAAGTGG
Exon 4	CTTTGTACAAGTTGATAAGT
Exon 5	CTCGTCTAAAAAGGTTTGTC
Exon 6	TAAACACAAATGCGGCTGCT
